# The Worm Turns

**Published:** 1896-08

**Authors:** 


### THE WORM TURNS.

"Declined without thanks." "Beg to be excused." "Not any for Joe," or something similar will about express it. And, this result might have been expected; ought to have been expected; but it seems it wasn't. A moment's reflection should have shown the friends of the Association Bill that in whatever light the members individually and collectively, may regard the homeopath from a professional standpoint, most of them are gentlemen, and are entitled to courteous treatment. It might have been forseen that such language as was used in Dr. Brittains "resolution" would give offense. It was an invitation to join in a movement to secure medical legislation—a most questionable step—ethically considered—accompanied by a slap in the face. But, there was *not* a "moment's reflection." There was no reflection at all, so great was the haste, and so fiery the zeal to get a bill to "suppress quackery" (?) that all else was lost sight of.

The committee who framed the bill should have protected the homeopaths from this gratuitous insult. They framed the bill, giving homeopaths one-third representation on the board of examiners (eclectics one-sixth, and the regulars one-half), after lengthy correspondence with leading homeopaths, in which correspondence those of us on the outside supposed this ratio was agreed upon, but it now transpires that the homeopaths insisted on a separate board. When the bill was adopted, the committee and the Association were fully committed to these parties as allies, having invited their co-operation, and they should have been respected as such. But Dr. Brittain's resolution is an insult upon the face of it. It declares in effect that the Association, by stress of circumstances was forced to do a detested thing, and that while in the act of doing it they gagged and kicked. That the homeopaths are incensed is not at all surprising; that they should indignantly refuse the proposed recognition so ungraciously tendered, is also quite natural. But what sort of position does it put the State Medical Association in? Through a mistaken notion of expediency,—spurred on by what, by some is considered to be an entirely imaginary sense of obligation (for the State Association is not the public's guardian, and is not responsible for its health), they have humbled themselves into the dust, and have been spurned with contempt. They have, in effect gone to these people, cap in hand, and acknowledging themselves whipped in the contest for medical legislation, begged the assistance of their *ci devant* enemy in procuring legislation to protect their self-assumed ward—the public health—from the dangers of all *other* quacks, condoning the quackery and fraudulent practices of the proposed ally,—and their petition so humbly preferred has been spurned, because of a concomitant resolution. The resolution said, in effect, We have swallowed you as we would have done a dose of castor oil; it was necessary; but if any fellow, in his individual capacity, dares to swallow you, he thereby "lowers the dignity of the profession," and we will expel him. Is it to be wondered at that the homeopaths have resented it? They not only decline the proffered alliance, but have instructed their executive committee to refuse to hold any correspondence with the despised "allopaths."

We believe their disgust and anger are genuine; but the writer has pointed out through the JOURNAL, time and time again, the fact that the homeopaths do not desire any kind of legislation to regulate the practice, nor do they intend that there shall be any. The homeopath dreads an examination, and the very mention of the possibility of having to be examined creates a panic in their ranks. We have pointed out also the repeated declaration of these people that they will resist the passage of *any bill* emanating from the Texas State Medical Association. I say—the Association might have known the result; should have anticipated such answer as they have received, but, they didn't stop to think.

And now, gentlemen of the committee, and of the Association, what are you going to do about it? *Perforce*, per *necessitate* the clause giving recognition to homeopaths will have to be stricken out, and the fight, if made, will have to be made along the lines as heretofore (though McLaughlin says none but a fool or an enemy of the Association would do it). The homeopath is your implacable foe, uncompromising in his hostility, and the sooner this is recognized the better for the dignity of the profession. For his own part, the writer of these few lines was never an enthusiastic admirer of humble pie, and he regrets to see it forced upon his friends. We can only console ourself by the reflection that we "told you so," and advise a return to correct principles. Lie down with the hogs and get up with the fleas, is an old Scotch proverb. Let the homeopaths most severely alone. As homeopaths, the Association—the profession have nothing to do with them. When they attempt to practice by methods other than those they profess, compel them to come up to the standard of requirements fixed for all others who engage in regular practice. We can not see how or why the mere title "homeopath" confers any right to practice medicine, or any immunity from examination.

The writer thinks that the Association should cease its efforts at medical legislation—having been so often rebuffed, and let the petition for protection come from the people—if they want protection; or else, ask the legislature to pass an act defining the requisites to the privilege of practicing medicine, and let there be one standard for all. The title, "Regulate the Practice," in itself, excites antagonism; it is a misnomer; it is not sought by the regular profession to regulate the practice, but to prevent the abuse of the privilege by throwing around it certain legal restrictions; to have the law entrust the privilege to those only who are qualified for its exercise. Let us then ask for what we want, and no longer insist on a bill to regulate the practice. As the JOURNAL has repeatedly said—the legislature does not concede to the medical profession the right to regulate—anything.

